# CARING FOR HOMEBOUND VETERANS DURING COVID-19 IN THE US DEPARTMENT OF VETERANS AFFAIRS MEDICAL FOSTER HOME PROGRAM

**DOI:** 10.1093/geroni/igac059.633

**Published:** 2022-12-20

**Authors:** Chelsea Manheim, Maya Katz, Leah Haverhals

**Affiliations:** Rocky Mountain Regional VAMC, Aurora, Colorado, United States; Rocky Mountain Regional VAMC, Aurora, Colorado, United States; VA Eastern Colorado Health Care System, Rocky Mountain Regional VA Medical Center, Denver, Colorado, United States

## Abstract

The COVID-19 pandemic made older, homebound adults with multiple chronic conditions increasingly vulnerable to contracting the virus. The United States (US) Department of Veterans Affairs (VA) Medical Foster Home (MFH) program cares for such Veterans residing in private homes of non-VA caregivers. In this qualitative study, we assessed adaptations to delivering safe and effective healthcare during the early stages of the pandemic for Veterans living in rural MFHs, interviewing (n=37) VA MFH care providers at 19 MFH programs across the US. We identified themes reflecting adaptations to care provision, including care providers increasing communication and education to caregivers who prioritized Veteran safety. Caregivers coordinated increasing telehealth visits, applied creative strategies to mitigate social isolation of Veterans and themselves, and Veterans were prioritized for in-home COVID-19 vaccinations. Study findings illustrate the importance of clear, regular communication and intentional care coordination to ensure high quality care for vulnerable, homebound populations during crises.

